# Dropping out and moving on: A qualitative study of autistic people’s experiences of university

**DOI:** 10.1177/1362361320918750

**Published:** 2020-05-31

**Authors:** Eilidh Cage, Jack Howes

**Affiliations:** 1 Royal Holloway, University of London, UK; 2 University of Stirling, UK

**Keywords:** autistic adults, dropout, higher education, non-completion

## Abstract

Autistic people are at high risk of dropping out of university, but little research has examined this issue. Fourteen autistic people participated in semi-structured interviews examining their experiences at university and the reasons they had dropped out. Thematic analysis identified patterns in participants’ responses. Themes were categorised as systemic issues, challenges within university or life after dropping out. Systemic issues centred around accessing diagnosis, autism understanding, mental health and outsider status. Challenges at university included culture shock, becoming disengaged, lack of proactive support and perceived inevitability of dropping out. Finally, life after dropping out was characterised by processing of trauma and shame, and realisation of doing ‘what’s right for you’. Together, these themes suggest that many improvements could be made at universities, such as more proactive support and creating more accessible environments. Societal-level change is also needed to improve educational opportunities for autistic people.

**Lay abstract:**

Many autistic people now go to university, but many of them also drop out of their studies. In fact, it is believed that autistic people are at higher risk of dropping out, but little research has been done to understand why this is happening. This research used interviews to take an in-depth look at 14 autistic people’s experiences of dropping out of university. All the things the participants talked about were examined closely by the researchers who identified common themes in what the participants discussed. The first set of themes captured some overarching issues faced by autistic people, such as difficulties with getting diagnosed, a lack of autism understanding, mental health challenges and feeling like an outsider. The next themes were organised within challenges faced at university, including a feeling of culture shock, becoming disengaged from one’s studies, a lack of proactive support from their university and a feeling that dropping out became inevitable. Finally, there were themes about life after dropping out, which involved a sense that the experience at university had been traumatic and shameful, but they believed people had to do what is right for them. All of these themes suggest that universities need to be better at supporting autistic people when they first come to university, and that they should actively offer clear support throughout and try and make the university environment more accessible for everyone, to ensure more autistic people have a positive university experience.

In 2017/2018, there were 11,015 autistic people studying across all years of study in the United Kingdom (Higher Education Statistics Agency, 2019). While statistics on autistic people dropping out of university are scarce, it is believed they are less likely to complete their studies than their non-autistic peers (Anderson et al., 2017; Newman et al., 2011). Yet, little is known about autistic people’s experiences of dropping out of university. It is vital this topic is understood since employment outcomes are poorer for autistic people (Allen & Coney, 2018; Friedman et al., 2013; Hurlbutt & Chalmers, 2004; Taylor & Seltzer, 2011) and higher education (HE) could help employment prospects (Chen et al., 2015; Migliore et al., 2012; Ohl et al., 2017). Due to the lack of research on this topic, this study used qualitative methodology to understand why some autistic people are unable to complete university.

Previous literature has examined autistic people’s experiences while they are at university, aiming to understand how universities can become more accessible (e.g. Chown, Baker-Rogers, et al., 2017; Jansen et al., 2016; Thompson et al., 2019). Factors promoting success for autistic people at university include determination and perseverance (Ward & Webster, 2018), powers of observation and passion for a topic (Barnhill, 2014; Van Hees et al., 2015). However, other autistic characteristics can present challenges, such as social interaction difficulties and sensory sensitivities (Knott & Taylor, 2014; Van Hees et al., 2015), which impact interactions with peers and studying in certain environments. Thus far, research has focused on understanding university experiences for autistic students without considering non-completion.

Many reasons may influence whether an autistic person is able to complete their studies. In the non-autistic population, identified factors include poor course choice, educational background, financial issues, a lack of social support and isolation (Bernardo et al., 2016; Christie et al., 2004; Davies & Elias, 2003; Harrison, 2007; Smith & Naylor, 2001). Tinto (1997, 2006; Tinto & Pusser, 2006) places the onus on the institution, theorising that to ensure retention, universities must offer appropriate support, outline clear expectations and have an environment which promotes social and academic integration. Some literature has examined completion for disabled (non-autistic) students, with low self-esteem, executive function difficulties, and poor academic and social integration related to non-completion (Duquette, 2000; Wolf, 2001). Little research has looked at non-completion specifically for autistic people, although Gurbuz et al. (2019) noted that current autistic students had more thoughts about withdrawing than their non-autistic peers. The current study follows a quantitative survey which examined factors that could contribute to non-completion for autistic people (Cage et al., 2020). The study found difficulties with the transition to university, low social and organisational identification and poorer academic experiences were factors contributing to non-completion. To the best of our knowledge, no other studies have examined this topic in autism, and none have used qualitative methodology.

Qualitative research enables interpretation of autistic people’s experiences and can provide invaluable insight into their lives, but this methodology is frequently undervalued (Bölte, 2014; Braun & Clarke, 2019). Given the paucity of research examining why autistic people are at higher risk of dropping out, qualitative research can generate new possibilities for future research. Critically, an autistic and non-autistic researcher worked together to design and conduct this study, a participatory approach which has been growing (e.g. Chown, Robinson, et al., 2017). The research question for this study was ‘*what are the experiences of autistic people who have dropped out of university?*’

## Methods

### Participants

Fourteen autistic participants from the United Kingdom participated in semi-structured interviews. Inclusion criteria for the study were that participants had studied in the United Kingdom, had experience of dropping out and an autism diagnosis. Mean age at time of interview was 38.5 (standard deviation (*SD*) = 9.04, range: 21–54), and there were seven males and seven females. Most participants were White British (*n* = 10) or another White background (*n* = 4). Most were diagnosed after they had dropped out of university (*n* = 10), one was diagnosed during and three beforehand, with mean age of diagnosis 31.33 (*SD* = 12.22).

Table 1 shows further information on the university context for each participant, such as where they had lived and when they had dropped out. Table 1 also details participants’ current education and employment status, with some subsequently returning to study.

**Table 1.** Participant details including gender, age, diagnosis timing and information on university at the time of dropping out.

Participant pseudonym	Gender	Age at interview	Employment status	Highest educational qualification	Diagnosis timing – before, during or after dropping out	Further university details at the time of dropping out
Finn	Male	39	Employed full-time	Other qualifications	After	Went straight after A-levels, dropped out twice after transferring between first years and lived in private accommodation.
Alexander	Male	38	Unemployed/unable to work	Other qualifications	During	Straight from college, lived in student accommodation and dropped out final year.
Angelica	Female	26	Employed full-time	2+ A-levels	After	Straight from sixth form, lived in student housing leased by the university and dropped out second year.
Angus	Male	47	Employed full-time	Higher national diploma	After	Went from college into second year of degree, lived with grandmother and transferred to first year then dropped out.
Harry	Male	21	Student	Scottish Highers	After	Took year out after school before university, lived in student accommodation and dropped out first term of first year.
Lydia	Female	49	Employed full-time/self-employed	Undergraduate degree	After	Straight from school, lived in student accommodation and dropped out first term of second year.
Heung-Min	Male	45	Unable to work/student	Undergraduate degree	After	Mature student, lived in private shared house, repeated first year and dropped out second year.
Siobhan	Female	34	Self-employed/student	Masters	After	Straight from college initially, dropped out of three degrees at different universities; lived in student accommodation twice and parents once; and dropped out twice from first year, once from second year.
Arya	Female	43	Unemployed	Undergraduate degree	Before	Mature student, dropped out of Masters, lived at home and dropped out first term.
Mauricio	Male	29	Employed full-time/self-employed	One A-level	Before	Straight from college on same campus, lived in supported living and dropped out second year.
Kelly	Female	37	Student	PGCert	After	Straight from school, lived in private accommodation with partner and dropped out first year.
June	Female	38	Student	Undergraduate degree	After	Straight from sixth form, lived in student accommodation and dropped out second year.
Nora	Female	39	Carer	Masters	After	Straight from school, lived in student accommodation and dropped out first year.
Mousa	Male	54	Employed part-time/self-employed	Masters	Before	Mature student, dropped out of Masters, lived at home and left after second term.

Pseudonyms are provided for each participant.

For recruitment, non-probability sampling was used with both voluntary and convenience sampling – participants had either previously consented to be contacted about research with the first author (E.C.) or responded to advertisements via social media, the mailing list ‘autism practitioners in HE’ or word-of-mouth. These sampling methods were utilised to efficiently reach potential participants who met the inclusion criteria within a short time frame. All participants gave full informed consent, and ethical approval was obtained from Royal Holloway, University of London.

### Materials and procedure

Semi-structured interview questions were developed following reviewing of the literature, a quantitative survey (Cage et al., 2020) and one researcher drawing on lived experience as an autistic person who had dropped out of university. Topics included transitioning to university, social experiences, academic experiences and support, and reasons for dropping out.

After reading information about the study and giving consent, all participants completed an online questionnaire which established their preferred mode of communication, availability for the interview and demographics (e.g. age, gender). Participants could view the questions before the interview; 10 participants elected to do so. Different modes of communication were offered (as detailed below) to allow flexibility and cater for diverse communication preferences (Vincent, 2019). Interviews were arranged at a convenient time in the preferred mode – seven participants completed the interview over the phone, Skype or in person, two used instant messaging on Skype and five used email. For email interviews, questions were sent via email and followed up with subsequent emails to seek clarifications and further information. All interviews occurred in May 2019 and took on average 46 min to complete (range: 35–75 min) if conducted verbally. Verbal interviews were audio recorded and transcribed verbatim. All participants were sent their transcripts and given the opportunity to amend these. Participants were reimbursed £15 for taking part, which they could accept in cash, voucher or as a donation to charity.

### Design and analysis

This study used experiential thematic analysis to identify patterns within participants’ experiences of dropping out. This type of analysis was selected as it effectively identifies meaning in participants’ descriptions of their experiences and thus suited the research question. The analysis was inductive through the identification of themes from the data. A contextualist approach was used, whereby both essentialism and constructivism are considered when attempting to understand participants’ experiences in terms of their realities and the wider impact of social context (Braun & Clarke, 2006). Themes were identified at the semantic level, based closely on participants’ accounts (Braun & Clarke, 2006). Guidelines for thematic analysis outlined by Braun and Clarke (2006, 2013) were followed: this included data familiarisation, generation of initial codes, theme searching, theme reviewing, theme defining and producing the report. NVivo 11 was used for coding, and both authors agreed on all themes.

### Author positionality

In qualitative research, it is vital that the positionality of the researchers is acknowledged and discussed (Braun & Clarke, 2019). E.C. is a non-autistic autism researcher who works in HE in the United Kingdom. She has an undergraduate degree and PhD in Psychology but also identifies with sociological viewpoints. She received her formative training during her PhD at the Centre for Research in Autism and Education, University College London (UCL), where principles of autistic involvement are part of their research ethos. Thus, the importance of including autistic people in autism research was a principle brought forward in the current research. Her research is generally about the experiences of autistic people within the context of a predominantly non-autistic world and trying to understand what can be done to support autistic people, particularly regarding their mental health. She has previously involved autistic people in her research, but this was limited to tokenistic involvement due to financial constraints. This study marks a turning point in achieving true collaboration, with the second author (J.H.) involved in all stages of the research.

J.H. is an autistic person who has personal experience of dropping out of university. He had an extremely unhappy time at university, struggling with living on campus alongside the socialising aspect of studying at university. This led to him dropping out after only one term, receiving a diagnosis shortly afterwards and therefore having a special interest in designing this research. The autistic author initially contacted the non-autistic author interested in getting involved and learning more about autism research in 2017. He is not affiliated with an academic institution, though works for an autism charity – his role in this research is in a completely independent capacity.

**Figure 1.**
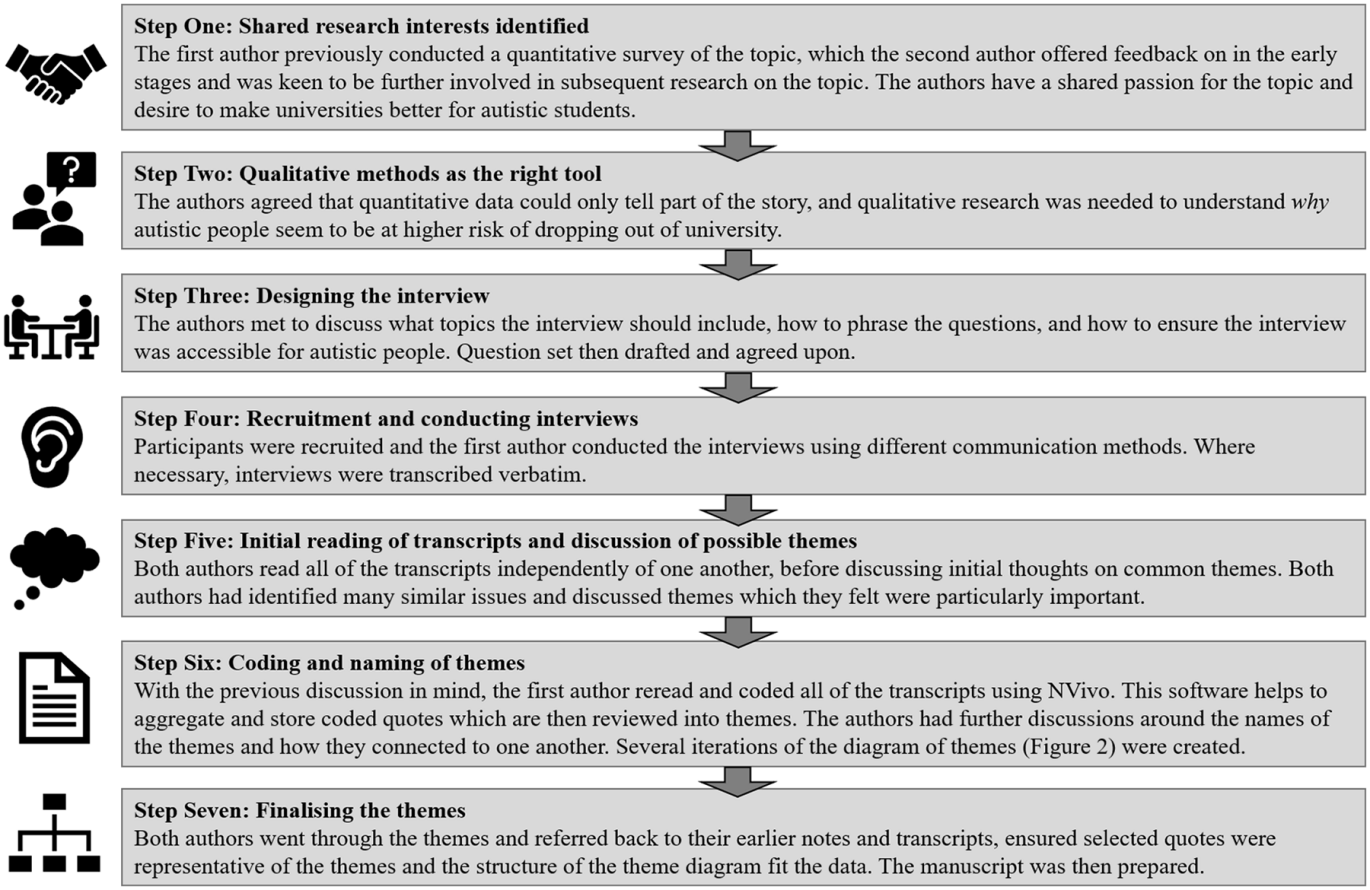
Diagram of the development of the research project and methodology.

After involvement in some of E.C.’s previous research and after several general discussions about research, we identified that we had shared interests and a desire to help improve universities for future autistic students. E.C. was able to use funding from her university to pay J.H. for his time, and we worked together to develop and conduct the current research, as outlined in Figure 1. In addition to coming from different backgrounds, we also have shared perspectives. We both lean towards a social model of disability rather than medical model (Shakespeare, 2006) and support the principles of neurodiversity (den Houting, 2018). These philosophies affect the data interpretation, as should be acknowledged (Braun & Clarke, 2019). The inclusion of both non-autistic and autistic perspectives enables us to analyse the participants’ experiences from both the ‘inside’ and ‘out’. For example, the autistic author brings his own personal experiences which influenced the formation of interview questions and the ability to analyse the themes from an autistic perspective, while the non-autistic author brings the practical experience of conducting research and knowledge of current systems within HE. Together, the positions are complementary, and we believe strengthen the quality of this research.

## Results

**Figure 2.**
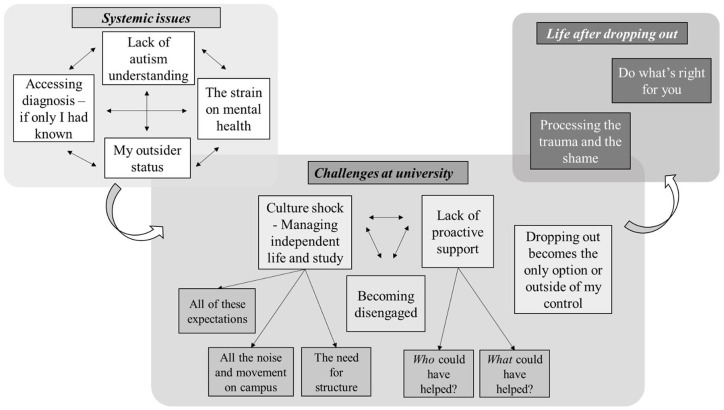
Themes and sub-themes within the three areas of systemic issues, challenges at university and life after dropping out.

Themes are organised as systemic issues, challenges at university or life after dropping out. Within each of these areas, themes and sub-themes were identified, as depicted in Figure 2.

### Systemic issues

Several themes were identified as systemic issues which reflected wider inter-related issues that had an impact on participants’ experiences.

#### Accessing diagnosis – if only I had known

Most participants had been diagnosed after dropping out, and a theme was identified that indicated difficulties in accessing diagnosis. ‘If only’ participants had known they were autistic, their experiences may have been different:

It would have been amazing if someone had figured out I was autistic and that is why I was struggling. (Nora)

I think had I had the diagnosis before, and known more about making the correct course choices, then I could have probably done it. I think that’s what I’ve taken from it, that I could have probably done it with the correct support. (Angus)

Some participants discussed barriers they had experienced accessing diagnosis as an adult or woman:

University was the first point I reached out and I went to the GPs and I got myself referred and every single healthcare professional that I met, and there was only three of them, and they all said, ‘you’re not a boy, you can’t have autism’. (Angelica)

#### Theme: lack of autism understanding

Participants felt that a lack of understanding and appreciation of autistic characteristics had prevented university from being accessible. When participants reflected on improvements to their experiences, they described the need for better understanding of autism and appropriate accommodations:

You want the sort of universities [that] make the autistic person feel comfortable, and they’ll be able to be honest and not feel guilty for being like ‘I can’t manage this’ or ‘I’m going to be a bit late for this deadline’. (Harry)

Other participants noted that although autism understanding needs improving, this did not necessarily mean specialist provision was required:

I didn’t need to be reminded I was autistic, nor did I want to be less autistic. Luckily [they] eventually stopped trying to change me and realised that while I was strange what I was doing was working [. . .] They started by trying to help me to be less autistic, but that was overall not helpful. I was there to study, not for someone to try and teach me social skills for the 27th time. (Mauricio)

#### Theme: the strain on mental health

Another theme highlighted how participants experienced mental health difficulties, often for several years beforehand, which worsened at university:

I didn’t feel happy [at university], but that was at least 75% due to my mental health being so poor – my PTSD became shockingly bad there, until I was too scared to lock my bedroom door at night in case I couldn’t get out. (Siobhan)

Circumstances at university built upon pre-existing mental health difficulties and left participants with limited resources to cope, often leaving participants overwhelmed:

I’d got myself into such a state about it, and then I just ended up having some sort of meltdown over it. And I think just the stress of it had been building and it’s such an intense feeling. The kind of response is to just run away and go well I just don’t want to feel like that again. So, I thought I just can’t do it [the degree]. (June)

I was really struggling to cope with it [the course], because I’m also a mum. And the course was actually affecting my ability, well the anxiety related to the course and the stress related to the course, was affecting my ability to function day-to-day in all aspects of my life. (Arya)

#### Theme: my outsider status

Participants described difficulties in navigating social spaces at university. Across these social difficulties, ‘my outsider status’ was identified as a theme. Despite trying to get involved, participants found themselves positioned as an outsider – a feeling many had felt prior and subsequent to university:

I just couldn’t fit in, the whole time I was there I was always just on the outside looking in at everyone else having the time of their lives, having the best experience they were ever going to have. I just wondered when that was ever going to happen to me. So yeah, for me it was just really cementing in my ‘you are an outsider’ status. (Angelica)

For those who lived with other students, they felt this environment particularly highlighted their outsider status:

It was the effort required to socialise with everyone [in halls] that was hardest of all, especially in [fresher’s] week, when everyone is expected to be out on the town. I did okay initially, going out once or twice, but I was only masking, and I could only do that for so long. So it wasn’t long before I started to become isolated within the group and an outsider, where I would remain for the rest of the year. (Alexander)

Others noted how their outsider status put them at a disadvantage compared with other students:

Now I realise that most of the other students were partly crowdsourcing their understanding – talking to each other and taking turns to ask questions of the lecturers, reporting back to each other. I wasn’t doing that at all, I was a little island of confusion in amongst all their networking, trying to figure it all out by myself. (Arya)

### Challenges at university

The next themes operated within the university experience, although are influenced by the issues outlined above.

#### Theme: culture shock – managing independent life and study

‘Culture shock’ was a theme, whereby university was described as an overwhelming change. This culture shock permeated living independently (where applicable) and independent study. For example, participants described how university study was different from their previous educational experiences, and they were left to their own devices. Elaboration of these points is noted within three sub-themes: ‘*all of these expectations*’, ‘*the need for structure*’ and ‘*all the noise and movement on campus*’.

##### Sub-theme: all of these expectations

Expectations played an important role in participants’ experiences, from the expectations placed on them by others, to expectations they placed on themselves and what they thought university would be like. These expectations often went unmet. For example, many had believed that university would enable them to study something they loved, find like-minded people or escape past difficulties:

The overriding thing that I felt was that I was just really disappointed. Like I always thought that university would be meeting people who were like me, and we’d be really politically active, and we’d stay up really late talking passionately about all these books that we’d read and stuff, and it wasn’t like that. (June)

Others also came to university not knowing what to expect, realising in hindsight that they were unprepared:

I didn’t really consider what halls there were, where it was located, etc. I sort of just went to the applicant day, I didn’t really take it in, I wasn’t really thinking about everything that was going to be happening and so although I turned up, I really hadn’t put much thought into it. (Harry)

One issue for many was feeling they did not know what was expected of them academically:

I really struggled to work out what exactly it was that they were asking me, and a few times I went and asked, ‘am I meant to be doing this or this?’ And I gave suggestions of what I thought that I was perhaps meant to be doing. But the feedback I then got was really mixed [. . .] so I was like ‘aaaah’. (Arya)

##### Sub-theme: the need for structure

Many participants described challenges with independent study. At the core of these difficulties, we identified a theme around the need for structure. University study felt unstructured and participants found it difficult organising their studies with little guidance:

I wasn’t prepared for the jump and the change, not just the level of study, because obviously it is harder, and you expect that. But what I found difficult was the amount of independent study and the fact we were supposed to be doing so much of it ourselves. (Angelica)

[University] was the biggest shock. I had worked out the formula for doing well in my A levels [. . .] Studying at undergraduate level was completely different. So much of my time was self-directed, and I had no idea what I was supposed to do with it. (Siobhan)

Lack of structure within courses with multiple elements also contributed to difficulties:

The thing with the course I was on, it was quite all over the place, you had everything from agriculture to chemistry to business to marketing. So, you had half a dozen subjects on the go, not particularly well linked. (Heung-Min)

##### Sub-theme: all the noise and movement on campus

The sensory environment significantly impacted participants’ ability to cope at university. Although participants had different sensory aspects that affected them, common sensory challenges were noise and processing movement of people:

The noise was impossible to filter out, and I couldn’t cope with the shared kitchen [. . .] I can’t figure out what people are doing in kitchens – you have to constantly predict movement and intentions, and I couldn’t, I felt like I was always getting in the way. (Siobhan)

So much going on, but there was a few bars at the union, and one of those was quiet, there was no blaring machines, no loud music or anything, and that’s where we tended to congregate. (Angus)

#### Theme: becoming disengaged

Another theme focused on disengagement from university, stemming from lack of motivation or structure in their studies. Some of this disengagement impacted participants submitting work or attending lectures:

I just couldn’t concentrate and did very little work. At school I was known as being clever and maybe I was scared of having to start over and prove myself. Plus I wasn’t that into my course choice, but didn’t know how to get out of it. I thought I would just throw myself into my studies to forget about everything, but I couldn’t seem to get started. (Nora)

As time went on my motivation dropped, to the point where I basically got nothing of substance completed at all towards my final-year dissertation. (Alexander)

Such disengagement was exacerbated when social or academic support was lacking:

I didn’t have any kind of support from friends or family, so the downward spiral of non-engagement and kidding myself things would work out okay began. (Finn)

#### Theme: lack of proactive support

Another theme centred around a dearth of proactive support. There were two features of this – that participants did not know *who* could help or *what* could have helped at the time.

##### Sub-theme: who could have helped?

In terms of who could have helped, participants described how they did not know who to talk to or that people they did talk to were unhelpful:

The head of the course, I had a tutorial [with him and] he wasn’t very polite, he was quite derogatory actually. And he was basically [saying] ‘can’t you bloody manage this’ and ‘can’t you bloody manage that’. [. . .] There was absolutely no one at any point really said to me in a nice, calming kind of way, ‘are you struggling?’ or ‘can we help you with something?’ No one looked further beyond the bare minimum they had to do for the student. (Heung-Min)

Some participants stated one solution might be to have a centralised point of contact:

[There needs] to be a point of contact for when autistic people are on the course, if they’re struggling with understanding instructions or they’re struggling with the structure and managing and organising their time. (Arya)

##### Sub-theme: what could have helped?

Participants described how they did not know what would have helped, although in hindsight several acknowledged their difficulties with introspection:

When she asked me what kind of support I need, I said I don’t really know because I hadn’t been to the university for a long time, I was coming back to it, and I didn’t know what they were offering or not. So I was more looking for her to suggest that this might be good or not. (Mousa)

For those diagnosed before or during university, they reported that the support they had received was inappropriate:

I was assigned someone to attend lectures with me and take notes for me. I was also given (essentially loaned, for free) a laptop to use so that I wouldn’t have to go out to work on a computer. [. . .] It was nice to have the support, but it wasn’t really the kind of support I needed [. . .] it was all a feeling of ‘too little, too late’, and not support in the right areas. (Alexander)

#### Theme: dropping out becomes the only option or outside of my control

Culmination of the previous themes contributed to participants’ feeling that dropping out became out of their control. After the build-up of the identified issues on participants being able to submit assignments or pass course requirements, dropping out was inevitable:

I think [academic support] could have made a difference for me, but I don’t think it would have been a major factor, I don’t think it would have made me stay and I don’t think it would have affected me moving forward. It might have got me into third year, but I don’t see the outcome being any different, I still would have walked away without a degree and gone ‘no, this isn’t for me’. (Angelica)

I never really wanted to drop out, but it got to the stage that there was no way back if I wasn’t going to get any work done. So it just sort of happened by default in the end. (Alexander)

### Life after university

Participants reflected on their lives after their experience of dropping out; we identified two themes in this context.

#### Theme: processing the trauma and the shame

For many participants, their experience was initially processed as something traumatic, often feeling like a failure, although with time some managed to process these feelings:

What annoys me, what I feel about it is it’s not that I’d failed the course after two years, what for me was the issue, was then I spent seven, eight, nine years worrying about that I’d failed that course and it really wasn’t worth it in the end, I should have just cut it loose. (Heung-Min)

I think more than anything it probably reinforced my negative self-talk about being a failure, I couldn’t even succeed at a third rate uni. (Kelly)

It’s almost like a glossed over kind of period for me where I pretend it wasn’t as bad as it was [. . .] It was pretty much the worst period of my life really, for me that was rock bottom. (Angelica)

#### Theme: do what’s right for you

Many participants realised dropping out had been the right option for them at that time, highlighting how important it was for others to do what is personally right in similar situations:

Perhaps this university course isn’t right for you, perhaps this university isn’t right for you. But it’s not a reflection on you, it’s got to be the right decision, it’s the right decision for you. (Angus)

Don’t blame yourself if it does happen – there are so many reasons university doesn’t work out for someone, and some of them are very fixable with time, experience, and more support. Lots of autistic people end up doing a lot better as mature students. (Siobhan)

## Discussion

This study qualitatively examined autistic people’s experiences of dropping out of university. The themes identified suggest several systemic issues influenced participants’ experiences, such as accessing diagnosis, autism understanding, mental health and outsider status. These systemic issues affected challenges presented at university. Here, we identified themes centred on feelings of culture shock, becoming disengaged, a lack of proactive support and dropping out being outside their control. After dropping out, participants reflected on the processing of trauma and shame, but also realisations around doing ‘the right thing’. This study makes an important contribution in understanding the challenges faced by autistic people at university and highlights potential means of reducing the risk of non-completion.

Four inter-related systemic issues impacting on participants’ university experiences were identified. First, many participants reported difficulties with accessing diagnosis, wishing they had known earlier that they were autistic. Obtaining an autism diagnosis, especially in adulthood, can come with delays and biases against highly verbal individuals or females (Jones et al., 2014; Lewis, 2017). However, this assumes diagnosis leads to support, which is not necessarily true – participants in this study who *were* diagnosed before university did not feel they had received adequate support. The other systemic issues identified suggest that having a diagnosis does not guarantee graduation.

Accordingly, another systemic issue was a perceived lack of autism understanding. Universities, staff and support are likely influenced by poor societal understanding. Past research has identified that autistic adults often do not feel accepted by others (Cage et al., 2018), and although knowledge of autism is reasonable (e.g. Dillenburger et al., 2013), underlying stigmatising attitudes can prevail (Cage et al., 2019; Kelly & Barnes-Holmes, 2013). An environment designed to suit and understand the needs of autistic people can benefit *all* students – a principle related to Universal Design (McGuire et al., 2006; Roberts et al., 2011). Universities have an opportunity to lead the way in creating accessible environments.

A third systemic issue was the exacerbation of mental health difficulties at university. Mental health difficulties are highly prevalent for autistic adults (Lever & Geurts, 2016) more so than in the non-autistic population although poorly understood (Howlin & Magiati, 2017; Lai et al., 2019). It is likely mental health difficulties impact on university experiences for autistic students irrespective of whether they are considering dropping out or not (e.g. Gelbar et al., 2014; Van Hees et al., 2015; Ward & Webster, 2018). Ultimately, appropriate mental health support for autistic people is desperately needed (Lake et al., 2014), within university and beyond.

The fourth systemic issue centred on perceptions of an outsider status. Autistic people do have social differences but can be socially motivated (Jaswal & Akhtar, 2019). Other autism research identifies feelings of being an outsider (Griffith et al., 2012; Lewis, 2017). As noted by participants, this outsider status prevented them benefitting from social learning opportunities and exchanging knowledge with other students. It is not purely the responsibility of the autistic person to be socially involved – non-autistic people must create environments where autistic people do not feel like outsiders. Furthermore, the facilitation of autistic peer support groups at university could be beneficial, with autistic students supporting one another (MacLeod, 2010).

These systemic issues influenced the themes which operated within university. We identified a theme of ‘culture shock’ in relation to independent life and study, with three sub-themes. Expectations from others and oneself often went unmet. Differences in expectations and reality is also noted as a theme for non-autistic students who did not complete (Harrison, 2007). There was also a need for structure and the shock of a lack of structure at university. This need may link to autistic characteristics and differences in executive functions (Kenworthy et al., 2008) and in the disability literature, Wolf (2001) suggested executive functions play a role in non-completion through issues with planning, forward-thinking and shifting between tasks. Furthermore, the sensory environment at university was described as overwhelming. Universities are less aware of sensory characteristics (Knott & Taylor, 2014), and current autistic students describe the risks of sensory overload in the university environment (Gurbuz et al., 2019; Van Hees et al., 2015). Further exacerbation of these issues occurred within the context of living with other students.

To reduce culture shock, helping autistic students develop realistic expectations through transitional programmes could be beneficial, by giving previews of university life (Lei et al., 2018; Toor et al., 2016). To help support autistic students who prefer more structured learning, academic staff should provide clear guidelines, share lecture materials in advance and use routines (Gobbo & Shmulsky, 2014; Hart et al., 2010). Universities should be open to adaptations of the sensory environment (Robertson & Ne’eman, 2008) as well as training staff to understand all aspects of autism. Relatedly, where an autistic student needs time and space to process, flexibility in degree structures may be beneficial. In a study of Italian university reforms which made university degree structures more flexible (as well as the provision of more support services), Di Pietro and Cutillo (2008) found a decline in dropout in the general student population. As before, this suggests principles that benefit autistic students benefit *all* students.

We also identified another theme centred on ‘becoming disengaged’. This disengagement may manifest itself in a lack of motivation or difficulties producing work. Universities should notice issues early and discuss with students what may be underlying the disengagement (which could relate to the issues identified above). Non-autistic participants in Harrison’s (2007) study of UK students who withdrew in first year also described gradual disengagement. It is important that universities acknowledge their role in ensuring engagement, for example, through regular feedback, supportive tutors and appropriate expectations of their students (Tinto & Pusser, 2006).

These themes linked to a lack of proactive support. We identified two sub-themes – not knowing *who* could have helped or *what* could have helped. Many did not know who they could have talked to or found staff unapproachable. They described the need for one centralised contact, as expressing their difficulties to many different people was exhausting. The difficulty with not knowing what could help may link to introspection difficulties (Bird & Cook, 2013). Again, the onus should lie with universities in ensuring they proactively offer appropriate support (Tinto & Pusser, 2006) and that staff have good understanding of autism (Barnhill, 2014). In the non-autistic population, positive relationships with tutors are important for helping students complete their studies (Bernardo et al., 2016).

Together, these themes linked to dropping out becoming the only option or outside of participants’ control. This finding implies that earlier intervention or more proactive support is needed to avoid this inevitability. However, it is worth noting the theme of ‘do what’s right for you’ as participants reflected on their experiences. They also described their experiences as traumatic with long-lasting feelings of shame. It is important to acknowledge that university may not work for everyone or be suitable at that point in their lives – for some, withdrawal (or interruption) may be the best option. Graduating from university also does not guarantee positive employment prospects – with increasing qualification level, autistic people are *less* likely to be employed (Allen & Coney, 2018). This issue may relate to some of the systemic issues around autism understanding and inclusion within society (Baldwin et al., 2014).

### Limitations

The sample consisted of predominantly late-diagnosed autistic people, who were undiagnosed at the time of dropping out. It is not possible to state whether there is a higher risk of dropping out for those undiagnosed or a recruitment bias. Furthermore, many of the participants had attended university when provision may have been poorer than today, whereby autistic students now have greater rights when studying (e.g. *Equality Act 2010*). However, given the lack of literature on non-completion for autistic students, this qualitative study adds value. As with all qualitative research, generalisability is not possible (Braun & Clarke, 2019) – the themes identified are based only on these participants. There was also no direct comparison to non-autistic people, but research on non-autistic dropout is extensive, which enables comparisons with the literature. This study highlights some unique issues associated with being autistic, and while there are some comparable elements (as discussed), factors cited as reasons for non-completion in non-autistic people, such as poor course choice or financial difficulties (Davies & Elias, 2003; Harrison, 2007), were not identified in this study. Models developed within the non-autistic population still have utility, such as Tinto’s (1997, 2006; Tinto & Pusser, 2006) approach which places the responsibility on universities rather than student for avoiding dropout.

To avoid dropout, universities must be proactive in supporting autistic students, provide support early on, enable better transitions and ensure support options are clear. Furthermore, working towards the principles of Universal Design, whereby universities are accessible for *everyone*, could mitigate issues for undiagnosed autistic people at university. Universities have a duty to ensure autistic people have equal opportunity to complete their studies wherever possible.
